# Medical Electricity

**Published:** 1887-08

**Authors:** 


MEDICAL ELECTRICITY. A Practical Treatise on the Applications of Electricity to Medicine and Surgery. By Roberts Bartholow, M. D. Third edition, enlarged and improved. Philadelphia: Lea Brothers & Co.

Like all of Prof. Bartholow's books, this supplied a much-needed want, and the call for a third edition shows the demand has not ceased. It is emphatically, as the author says, a book for the student and practitioner. It is written from a physician's standpoint, and furnishes the working knowledge necessary to intelligently use electricity as a therapeutic agent. So used, and not vaunted as a cure-all, there is no doubt of electricity's usefulness—nay, far more, often of its indispensability. In this edition much new matter has been added, making a volume which, like Prof. Bartholow's lectures, is always interesting and instructive. It seems strange that under electrolysis there should be no mention of the use of this means to remove superfluous hairs. H. H.

